# Assessing chemotherapy dosing strategies in a spatial cell culture model

**DOI:** 10.3389/fonc.2022.980770

**Published:** 2022-11-24

**Authors:** Dhruba Deb, Shu Zhu, Michael J. LeBlanc, Tal Danino

**Affiliations:** ^1^ Department of Biomedical Engineering, Columbia University, New York, NY, United States; ^2^ Data Science Institute, Columbia University, New York, NY, United States; ^3^ Herbert Irving Comprehensive Cancer Center, Columbia University, New York, NY, United States

**Keywords:** chemoresistance, breast cancer, doxorubicin, metronomic chemotherapy, heterogeneity

## Abstract

Predicting patient responses to chemotherapy regimens is a major challenge in cancer treatment. Experimental model systems coupled with quantitative mathematical models to calculate optimal dose and frequency of drugs can enable improved chemotherapy regimens. Here we developed a simple approach to track two-dimensional cell colonies composed of chemo-sensitive and resistant cell populations *via* fluorescence microscopy and coupled this to computational model predictions. Specifically, we first developed multiple 4T1 breast cancer cell lines resistant to varying concentrations of doxorubicin, and demonstrated how heterogeneous populations expand in a two-dimensional colony. We subjected cell populations to varied dose and frequency of chemotherapy and measured colony growth. We then built a mathematical model to describe the dynamics of both chemosensitive and chemoresistant populations, where we determined which number of doses can produce the smallest tumor size based on parameters in the system. Finally, using an *in vitro* model we demonstrated multiple doses can decrease overall colony growth as compared to a single dose at the same total dose. In the future, this system can be adapted to optimize dosing strategies in the setting of heterogeneous cell types or patient derived cells with varied chemoresistance.

## Introduction

Chemotherapy dosing strategies have primarily followed a maximum tolerated dosage (MTD) approach, in which patients are given high doses of chemotherapy to kill as many tumor cells as possible (1–5). This approach is based on early assumptions that tumors are composed of a homogenous, exponentially growing cell population and thus maximum doses are likely to cause the highest disease eradication (6, 7). However, tumors are composed of genetically heterogeneous cells with varied chemoresistance, and further diversity in chemoresistance can arise due to drug selection pressure from traditional high dose treatments (8–10). Thus, while eradicating chemosensitive cells, the MTD approach can lead to faster growth of chemoresistant cell populations, leading to faster relapse and eventually worse outcomes (11). Furthermore, due to the severity of side-effects at high doses, administration of chemotherapy using the MTD approach is typically separated by timescales as large as weeks to allow normal tissue to recover (12). In contrast to MTD, metronomic chemotherapy (MCT) (13, 14), where chemotherapy is delivered at lower doses more frequently, has been explored and demonstrated in some cases to be a more optimal dosing strategy in terms of both measures of efficacy and resistance development (15–18).

Since chemotherapeutic dosing schedules are often determined empirically in clinical trials, the discovery of optimal dosing strategies such as MCT for individual drugs has been limited. Several in silico studies coupled with *in vitro* assays have demonstrated that intra-tumor heterogeneity and dosing strategy can affect tumor response to chemotherapy (9, 15, 19, 20). However, *in vitro* drug efficacy assays are typically performed on homogenous, chemosensitive tumor cells, which fail to incorporate tumor spatial heterogeneity and thus are not ideal for investigating the spatial competition and dynamic interaction between drug sensitive and resistant cell subpopulations in response to chemotherapy. Inevitably these models are not predictive of drug efficacy or dosing schedules, particularly in patients who have developed drug resistance from previous treatments (6, 21). In order to recapitulate the complexity of the tumor microenvironment, three-dimensional (3D) multicellular spheroid and organoid models have been employed to incorporate transport dynamics and intricate cell-cell interactions that are naturally presented *in vivo* (20, 22–24). However, it can be challenging to incorporate clonal heterogeneity in a spatially-controlled manner or visually track resistant clone trajectories in 3D cell culture models. The effects observed in 2D models may not necessarily be consistent with those in 3D models and depend on the correlation varies based on cell/cancer type. Therefore, simplified model systems in 2D can enable the development of initial hypotheses that should then be used in concert with additional assays.

Here we use a 2D culture model to track dynamics of chemo- sensitive and resistant cell populations in a growing colony. The colony expansion can be tracked for at least three weeks in standard tissue culture wells, making long-term analysis of chemotherapy dosing schedules possible. We use this system to control parameters including the initial resistant cell proportion as well as dose and frequency of drug delivered. We then build a simple mathematical model and study the effect of various chemotherapy dose and frequencies on colony growth. Finally, we validate the findings of the mathematical model in an *in vitro* model.

## Materials and methods

### Development of chemoresistant breast cancer cell lines

The mouse triple negative breast cancer cell line 4T1 was obtained from ATCC (RRID : CVCL_0125) and was cultured in RPMI cell culture medium (RPMI 1640, Thermo Fisher, MA) supplemented with 10% fetal bovine serum (Thermo Fisher, MA) and penicillin-streptomycin (100 IU/ml). Cultured cells were maintained in a controlled humidified atmosphere of 5% CO_2_ in air at 37°C. Cells were subcultured every 3-4 days when reached over 80% confluency. Drug sensitivity of wild type cells were evaluated using a colorimetric cell proliferation assay (MTT assay, V13154, Thermo Fisher, MA) and a dose-response curve was constructed by culturing cells and evaluating cell viability in the presence of various chemotherapy drug concentrations. In order to establish chemoresistant cell lines, wild type cells were initially cultured in the presence of a very low concentration of chemotherapeutic drug (approximately 5% of IC50). Cell medium was replaced every 2-3 days until 75-80% confluency was reached. Cells were subsequently detached and subcultured and the viability of collected cells was evaluated using trypan blue staining. Drug concentration in the subcultured cells was doubled when over 90% of collected cells were viable. Otherwise, cells were subcultured and maintained at the same concentration for additional 1-2 passages until desired viability was achieved. Cell drug sensitivities were evaluated at each passage using MTT assay and cells with selected drug resistance were cryopreserved. In the present work, the resistance level of cells is defined as the highest chemotherapy concentration the cells were exposed to while being cultured. For example, the label 4T1-Dox [50 nM] means 4T1 cells that are able to replicate and grow in the presence of 50 nM doxorubicin (Sigma-Aldrich, MO) and the viability of cells at confluency are over 90%.

### 2D radial growth cell culture model

**Figure 1:**
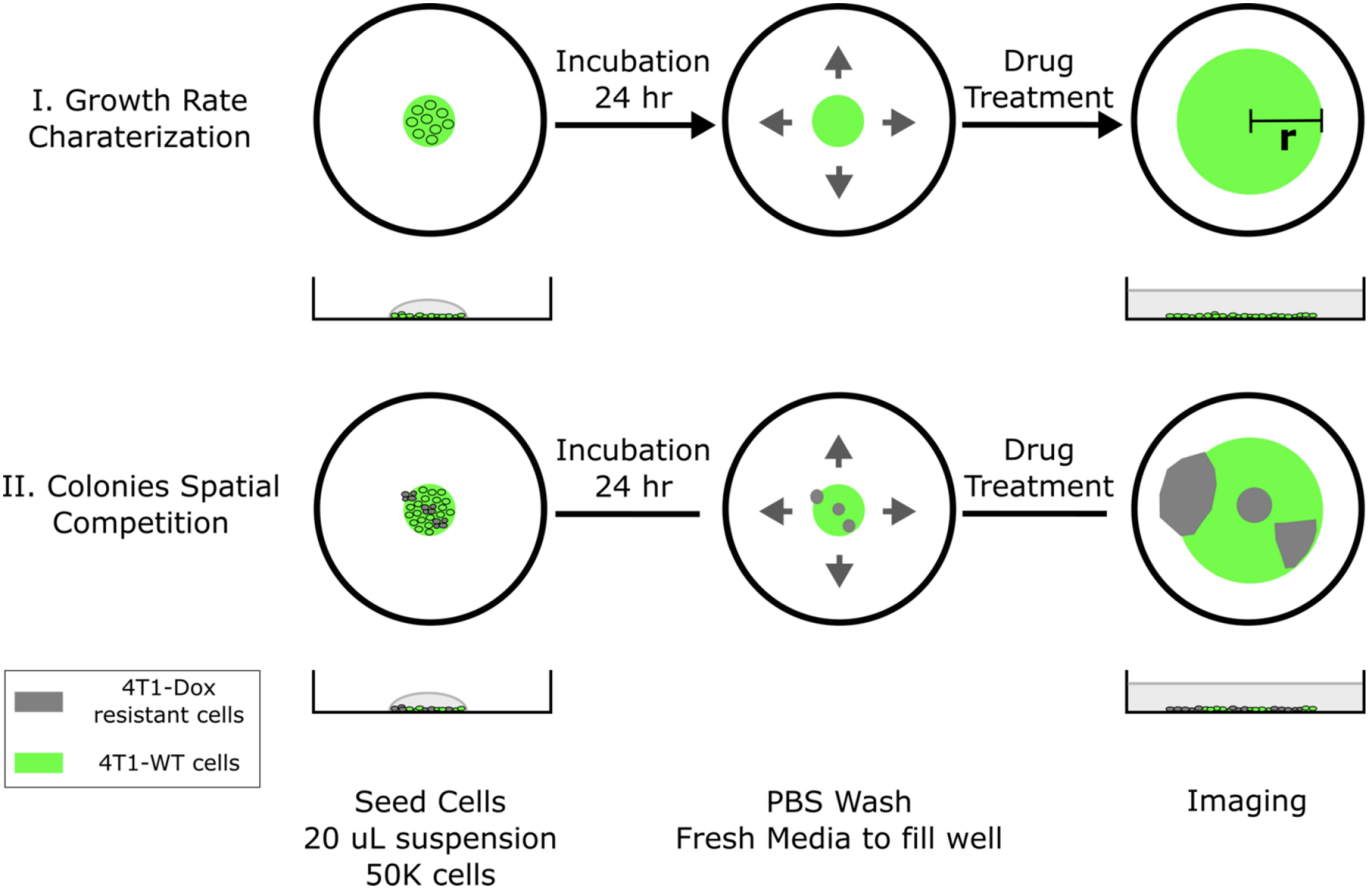
Schematic of 2D spatial colony system. (top, case I) Chemosensitive 4T1 cells are seeded in a well plate and grow radially, where colony radius and cell fluorescence are tracked over time. (bottom, case II) A mixture of chemosensitive and chemoresistant cells are premixed or spotted sequentially to establish a spatial competition model between cell populations.

Cryopreserved cells with desired chemoresistance were thawed and cultured in the presence of chemotherapeutic drug with matched concentration for at least 2 passages prior to 2D radial growth experiments for cells to reach stable growth state. Harvested cells were then resuspended in cell culture medium with a density of 1x10^6^ cells/mL. Single drops of 20 μL cell suspension were subsequently seeded in the center of each well on a surface treated 12-well plate. Significant spreading of the drop was not observed due to the hydrophobicity of treated well plate surface. Cells were allowed to settle for 24 hours under normal culture condition (5% CO2 in humidified air, 37°C) followed by a gentle PBS wash to remove dead or loosely attached cells. Fresh medium was then added to cover the whole well. At this point, a single colony of cells should have already appeared in the center of each well, the diameter of the colony is defined as the initial diameter and can be adjusted by varying the combination of seeding drop volume and seeding cell density. The seeded colonies would start expanding radially once the plates were returned to standard culture condition. Gentle PBS wash and refill of fresh medium was done every 24 hours (**Figure 1**, case I).

The cell seeding procedure can be modified to incorporate spatial heterogeneity in drug resistance across the colony. Single drops of 20 μL wild type 4T1 cell suspension (1x10^6^ cells/mL) were first seeded in the center of each well. Using a 10 μL pipette tip, a 5 μL drop of resistant cell suspension (1x10^6^ cells/mL) was gently added into the center of each seeded big drop without disturbing the big drop. The tip was inserted into the big drop vertically and the resistant cell suspension was slowly injected right above the bottom of the well. Cells were allowed to settle for 24 hours before a PBS wash. Fresh medium was subsequently added to fill the whole well to initiate colony expansion. The tip can be inserted into the center or near the edge of the drop and the resulted resistant subcolony will be observed at the corresponding location within the big colony (**Figure 1**, case II). The volume of added resistant cell suspension defines the size of resistant subcolony. It is also possible to incorporate multiple resistant clones by adding more drops of resistant cell suspension. In the case of coculturing chemosensitive and chemoresistant cells, 4T1-Citrine (fluorescent wild type 4T1 cells) were used to differentiate different cell populations.

### MTT assay

Cell viability assays were done according to manufacturer’s instructions (V13154, Thermo Fisher, MA). Briefly, cells were seeded on a 96-well plate with 100 μL of cell suspension with density of 6000 cells/well and the plate was incubated under normal cell culture condition for 24 hours leading to 50 – 60% confluence. 10 μL of MTT reagent was added into each well. The well plate was immediately returned to cell culture incubator and was incubated for another 3-4 hours until visible purple precipitations show up at the bottom. Another 100 μL of detergent reagent was then added to dissolve the purple precipitations. OD measurement of the well plate was done at 570 nm wavelength using a Tecan microplate reader (Thermo Fisher, MA) and the background for each well was measured at 690 nm wavelength and was subtracted from the readings. Wells with wild-type cells were controls and each condition was tested in at least triplicate wells.

### Image analysis

Each well was imaged using an EVOS FL Auto 2 Cell Imaging System (Thermo Fisher, MA) at desired time points. The scope and accessories were programmed using the Celleste Imaging Analysis software (Thermo Fisher, MA). Customized MATLAB code was used stitch subplot and generate images of each entire colony. Images were analyzed using Image J (NIH). Outlines of each colony were traced manually in ImageJ. Area measurements were used to calculate colony diameter assuming each colony is a perfect circle. To calculate the fraction of resistant cells in ImageJ, first, the background was removed by setting a pixel threshold. Next, the area occupied by the cells was calculated using the circle and analyze function in ImageJ. As all cells’ nuclei were stained with Hoechst stain (Invitrogen™ NucBlue™ Live ReadyProbes™, ThermoFisher, MA), and all wildtype cells were transduced to express Citrine (25), the fraction of resistant cells were obtained from the values of (Hoechst – Citrine)/Hoechst. The same background threshold was maintained for all images.

### Statistical analysis

In all *in vitro* assays, number of replicates were n = 3 or 4, and the standard error is calculated with standard deviation divided by the square root of n. Statistical significance was calculated by two-tailed, paired t-test while comparing two populations and was calculated by ANOVA with two-factor and replication comparing three or more populations.

## Results

### Doxorubicin-resistant 4T1 breast cancer cells in the 2D co-culture system dictate the overall response to doxorubicin treatment

**Figure 2:**
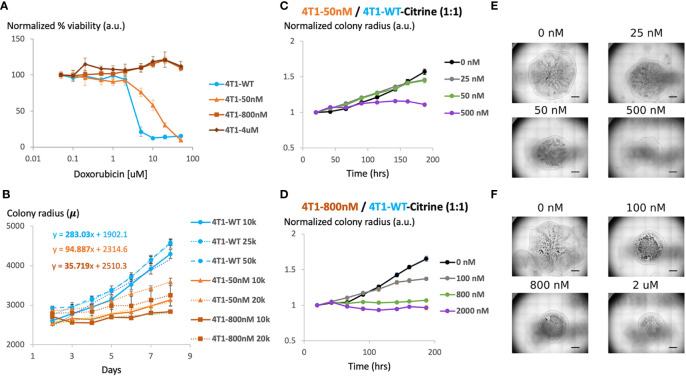
Generation and characterization of doxorubicin-resistant 4T1 cells in a 2D model system **(A)** Viability of chemosensitive or Dox-resistant 4T1 cells across varied Doxorubicin added to culture determined by MTT assay. Error bars represent standard deviation for 3 replicates. **(B)** Radial growth of chemosensitive and Dox-resistant colonies as a function of time for various seeding densities. **(C)** Colony radius normalized to starting radius in a 1:1 seeded mixture of sensitive and 50nM Dox resistant cells in the presence of 0 nM, 25 nM, 50 nM or 500 nM doxorubicin. **(D)** Colony radius normalized to starting radius in a 1:1 seeded mixture of sensitive and 800nM Dox resistant cells in the presence of 0 nM, 100 nM, 800 nM or 2 µM doxorubicin. **(E, F)** Stitched 4X brightfield images demonstrating size of colony and morphology across various seeding and chemotherapy conditions in **(C, D)**, respectively. Scale bar = 1000 µm. Number of replicates, n = 3, and the standard error is calculated with standard deviation divided by the square root of n.

MTT analysis confirmed that gradually increasing chemotherapy concentration in cell culture leads to the generation of 4T1 chemoresistant cell lines with different level of chemoresistance (**Figure 2A**). While the IC50 of wild type 4T1 cells was about 2 μM, the three representative resistant cell lines we created were able to maintain viability at much higher drug concentrations. For 4T1-Dox [4 μM], 4T1 cells that were cultured in the presence of 4 μM of Dox, we observed minimal to no reduction in cell viability even at 100 μM, which was the highest concentration tested due to the limited drug solubility.

We next assessed the effect of initial cell density on the growth of the colonies over time. Specifically, maintaining the seeded drop volume constant at 20 μL, increasing seeded cell number (all sensitive cells) from 10K to 50K resulted in an over 2x increase in initial colony diameter (**Figure 2B**). However, the colony expansion rates indicated by the change in colony diameter within a fixed time period (slope of the growth curves) were similar despite the difference in initial colony diameter. Reduction in serum level impeded colony expansion, however, where a 10x reduction in serum level was required to achieve a significant reduction in colony growth rate (**Supplementary Figure S1**). 4T1-Dox [50 nM] (which grow at 50 nM doxorubicin) colonies had a slower growth rate than the 4T1-Wt colonies and the 4T1-Dox [800nM] (which grow at 800 nM doxorubicin) colonies were the slowest.

During later cycles of chemotherapy in patients, intratumor heterogeneity is common and sensitive and resistant cells often co-exist (26). Hence, we hypothesized that our model system could be used to test the overall tumor response to chemotherapy. Here, we seeded single 20μL drops of mixed populations of 4T1 wild type and 4T1 resistant cells (total 1x10^6^ cells/mL) of total ~20k cells per colony. Mixing resistant and sensitive cells and seeding mixed cells lead to the formation of colonies with both cell populations and resistant cells were evenly scattered across the entire colony. To mimic the clinically relevant level and range of resistance (27), we chose to utilize cells with lower resistance levels (<1 μM) for our characterization. For colonies with cells that were less resistant (4T1-Dox [50 nM]), increasing the initial proportion of resistant cells had minimal impact on colony expansion in the absence of any doxorubicin treatment (**Figure 2C**). For all tested conditions, doxorubicin treatment had minimal impact on colony expansion during the initial stage while the reduction in colony expansion was more pronounced during later time points (**Figures 2C–F**). Mixed colonies of cells were able to grow while varying the inoculation location, percentage of FBS in the media and the fraction of resistant cells compared to that of the sensitive cells (**Supplementary Figures S2–S5**). Taken together, our data suggests that presence of doxorubicin resistant 4T1 cells along with sensitive cells can alter the overall response to doxorubicin in our model and this response varies based on the dose and time of the treatment.

### 2D co-culture system can be optimized to identify a critical drug concentration and to screen for various treatment regimens

**Figure 3:**
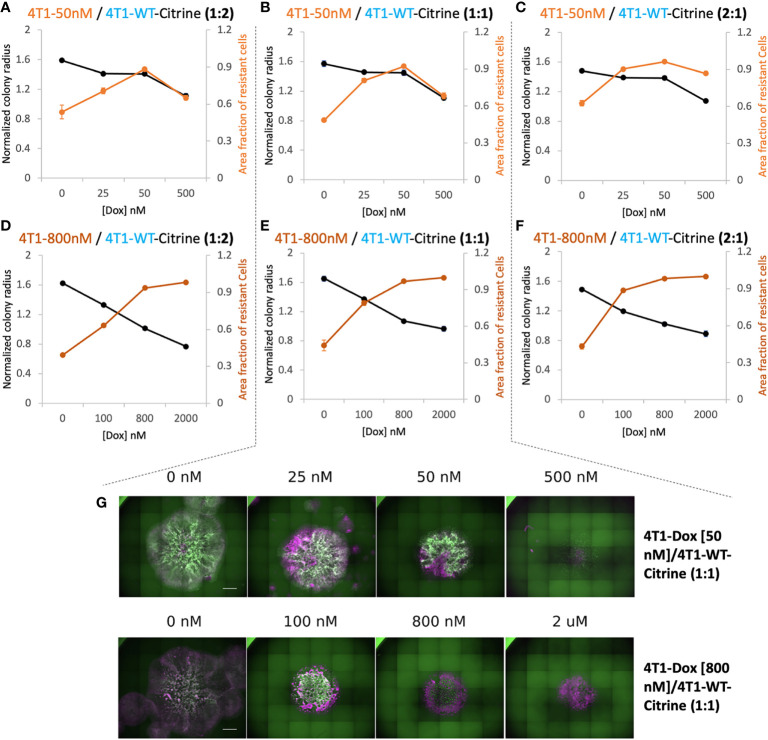
Relationship between overall 2D tumor colony size and treatment efficacy. **(A–C)** Normalized radius (black) and Area fraction of resistant cells (red) for mixtures of chemosensitive 4T1 and 50 nM 4T1-Dox resistant cells at varied ratios. **(D–F)** Normalized radius (black) and Area fraction of resistant cells (red) for mixtures of chemosensitive 4T1 and 800 nM 4T1-Dox resistant cells at varied ratios. The fraction of resistant cells was calculated from area of the mixed colonies occupied where all cells were stained with Hoechst and doxorubicin-sensitive cells expressing Citrine. Resistant fractions = (Hoechst – Citrine)/Hoechst. **(G)** Stitched 4X fluorescent images of colonies demonstrating the distribution of chemosensitive cells (4T1-WT, Citrine) in each colony (Hoechst) in the presence of doxorubicin. Colonies were formed by seeding 1:1 mixture of 4T1-WT and 50 nM resistant cells (top, corresponding to **(B)** or 1:1 mixture of 4T1-WT and 800 nM resistant cells (bottom, corresponding to **(E)**. Scale bar = 1000 μm. Number of replicates, n = 3 or 4, and the standard error is calculated with standard deviation divided by the square root of n.

In order to differentiate the response of sensitive and resistant cells we transduced the 4T1 wildtype cells to express Citrine fluorescent marker (25). Citrine labelled 4T1 cells retained their response to Doxorubicin similar to that of 4T1 wildtype cells (**Supplementary Figure S6**). Hoechst staining of each colony was carried out at the final time point. In the end, the whole cell population stains with Hoechst while only the sensitive wildtype cells show the Citrine signal. Hence, cells that were Hoechst-positive, but Citrine-negative were identified as the resistant cells. By measuring the occupied area of each cell population, we found high drug doses caused more reduction in final size of the colony but at a price of increased proportion of resistant cells in the colony (**Figure 3**). The drug concentration that led to a colony composition of equal amount of chemoresistant and chemosensitive cells can be considered as a “critical concentration”. Both the initial proportion of resistant cells as well as their level of resistance determined this critical concentration and it was much lower when majority of seeded cells were chemoresistant. Interestingly, when 4T1-Dox [50 nM] cells were seeded with lower or equal number of 4T1-WT cells, after critical concentration, the fraction of resistant cells decreased along with the decrease in total colony radius as a response to high concentration of Doxorubicin.

### A mathematical model based on the behavior and parameters of the 2D co-culture system can be used to predict cumulative chemotherapy regimens to attain the lowest volume of tumor

**Figure 4:**
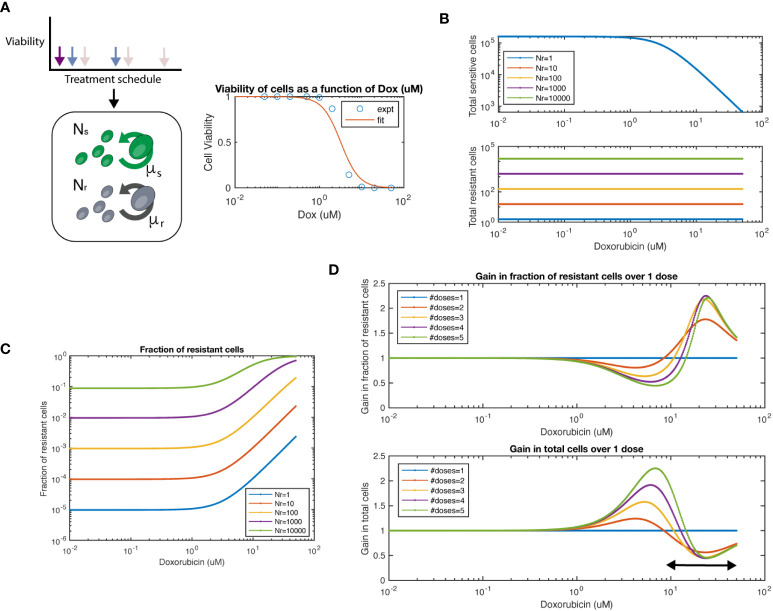
Mathematical model of chemosensitive and chemoresistant populations. **(A)** (left) Schematic of the underlying system modeled, whereby resistant (Nr) and sensitive (Ns) cell populations grow exponentially, and are then subject to chemotherapy regimens of varying frequency and dosage. (right) Viability of chemosensitive cells in response to doxorubicin. Data was fit to a Hill function with hill coefficient = 2, and EC50 = 3.2 μM. **(B)** Total sensitive cells and total resistant cells at the end of interval as a function of starting dosage. Colors indicate varied initial conditions of N_r_ = 10^0^, 10^1^, 10^2^, 10^3^, 10^4^, where N_s_ = 5x10^4^. **(C)** Fraction of resistant cells as a function of starting dosage for varied initial conditions. **(D)** For a 1:1 mixture of Ns to Nr, number of doses is varied. The fraction resistant, total cell population are plotted on the top row. The ratio of a particular number of doses to 1 dose is termed the “Gain”, both for fraction resistant and total cell population. The arrow at bottom panel shows the chemotherapy dose range where multiple doses show lower number of total cells over single dose.

To quantitatively understand the dynamics of chemosensitive and chemoresistant cells subject to different dosing strategies, we built a mathematical model. In this model, chemosensitive (N_s_) and chemoresistant (N_r_) cells are initially mixed at a given ratio and then assumed to exponentially grow at rates μ_s_ and μ_r_, respectively (**Figure 4A**). To subject cells to chemotherapy, we used our experimentally determined doxorubicin dose-response viability curve for wildtype and resistant 4T1 cells (**Figure 2A**). We assumed that resistant cells were not affected by chemotherapy at the tested doses, such as in the 4T1 (Dox 3uM) cell line we created experimentally. Thus, cells after a given interval of time would have the population level


$$N=V(x)*e^{a}$$

where V(x) is the viability value for cells at a particular dose x, and a is the growth rate (a scaled time interval of t=1 is assumed here for simplicity). In the case of multiple doses, each dose is fractionated across a smaller interval, but repeated for multiple intervals, keeping the same total time interval as the single dose. Thus, the population level following multiple doses (n) is


$$N=V{\left(\;x/n\;\right)}^{n}*e^{a}$$

(Supplemental material).

We first simulated the model and varied the number of resistant cells Nr from 10^0^-10^5^, while keeping the number of chemosensitive cells at 5x10^4^. As expected, we observed that the total number of chemosensitive cells was reduced with increased dose, while the amount of chemoresistant cells was not affected by chemotherapy dose (**Figure 4B**). We next calculated the fraction of resistant cells across multiple doses and initial conditions (**Figure 4C**). For high doses, such as in a maximum tolerated dose (MTD) approach, resistant populations eventually dominate the cell population as previously noted. Next, we set sensitive and resistant cells at a 1:1 initial ratio and set the growth rate of resistant cells as 20% of sensitive cells, as measured from data in **Figure 2C**. We found that when varying the number of doses applied, the fraction of resistant cells and total cell number as compared to a single dose (defined here as the “gain”) was a function of the initial starting dose of chemotherapy used (**Figure 4D**). At concentration ranges of ~1 - 10nM of chemotherapy resulted in populations with a lower fraction of resistant cells when comparing multiple doses to a single dose. In turn, multiple doses also resulted in larger total cell populations. However, at higher concentration ranges of more than ~10nM, the relationship was reversed. Higher levels of chemotherapy with multiple doses led to an increased fraction of resistant cells and smaller total cell number. As expected, the relationship between increasing resistant cell fraction and smaller total cell number was inversely proportional, such that there was no particular number of doses or starting dose that would allow for beneficial optimization of both measures.

We next tested if we could simulate the collateral effect of chemotherapeutic agents. To address this, we used our model to simulate the effects of multiple doses on healthy human mammary epithelial cells (HMEC). We extracted an IC50 for the HMEC line from a previously conducted study (28), which reported EC50 = 0.052 μM for Doxorubicin. This value is approximately 60-fold lower in terms of the EC50 of our 4T1 cell line (EC50 = 3.2 μM). We observed an approximate ~20% difference between single and multiple doses in terms of number of cells (**Supplementary Figure S7**, top panel). Interestingly only minor differences were shown between 2-5 doses. In this same regimen, we noted more than 2-fold reduction in tumor size (bottom panel). Thus, our model can simulate the trade-off in terms of tumor growth and collateral effects.

In summary, based on the kinetics of our 2D model system, this simplified mathematical model can predict a critical concentration of drug and a schedule of treatment to achieve the smallest cell populations, the lowest proportion of resistant cells, or in between points that balance the trade-off between these outcomes.

### 2D co-culture system can test the therapeutic benefit of multiple dosing compared to single dose

**Figure 5:**
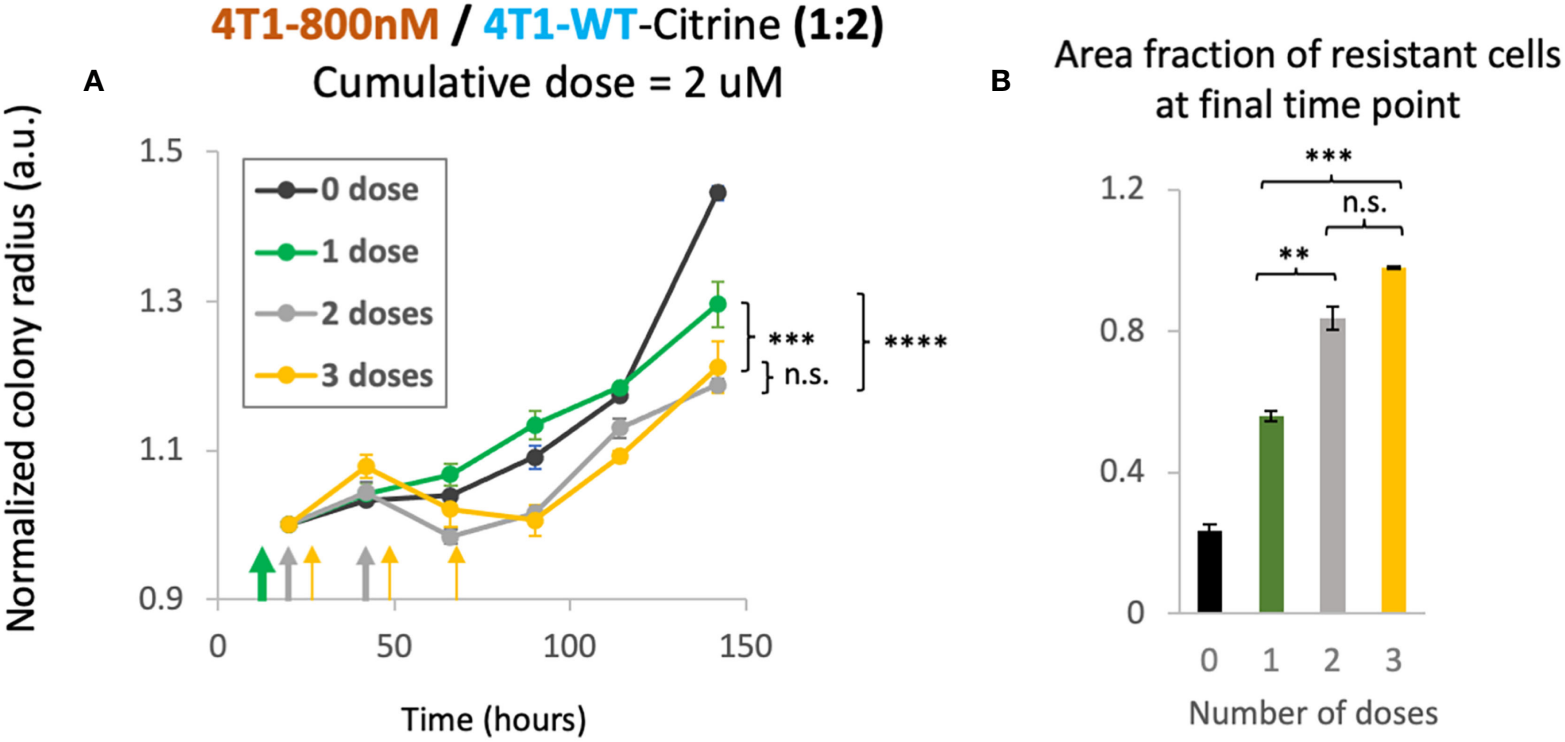
Cumulative treatment regime and long-term overall efficacy. **(A)** Normalized colony radius as a function for cumulative doses of 2 μM split into 1-3 administrations (green, grey and yellow arrows). Mixed colonies of 4T1-Dox [800nM] and 4T1-WT were seeded with a 1:2 ratio. Cumulative dosage for each chemotherapeutic schedule is defined as dosage level (in nM) multiplied by duration of drug treatment (dosing frequency [1/day] × n days, n = 1, 2 or 3). **(B)** Area fraction of resistant cells at final timepoint (142 hours) was calculated from area of the mixed colonies occupied where all cells were stained with Hoechst and doxorubicin-sensitive cells expressing Citrine. Resistant fractions = (Hoechst – Citrine)/Hoechst. Number of replicates, n = 3, and the standard error is calculated with standard deviation divided by the square root of n. 2-way Anova was carried out to calculate the significance in panel **(A)**, p value. ** = p<0.01, *** = p<0.003, **** = p< 2 x 10^-6^. n.s., not significant.

As our mathematical model suggested therapeutic benefit with small tumor sizes under multiple dosing, we investigated whether the schedule of drug treatment may alter the treatment efficacy in our *in vitro* 2D model. In order to study the effect of multi-dosage schedule on colony expansion, we first seeded 4T1-WT cells and treated the colony with 2 different chemotherapy dosing schedules. Specifically, we varied the drug concentration and the frequency of treatment per week. For example, to reach a cumulative dose of 600 nM, we utilized 1 dose of 600 nM, 2 doses of 300nM or 3 doses of 200nM of doxorubicin. In this way, a cumulative treatment of 600 nM per week was achieved in each scenario. Gentle PBS wash was done several times after each dose to remove waste in culture and fresh media with drug was then added for the next dose. We found that when holding the cumulative amount of doxorubicin constant, colony expansion kinetics were not affected by altered dosing schedule for the 4T1 wildtype colonies (**Supplementary Figure S8**). Next, we tested the colony expansion kinetics under varying dosage schedule for mixed colonies of 4T1-Dox [800nM] and 4T1-WT seeded with a 1:2 ratio. We chose this ratio to mimic a clinical situation of onset of resistance with fewer number of resistant cells than that of sensitive cells. We chose to achieve a cumulative dose of 2 μM that we tested previously in this ratio of mixed colonies (**Figure 3D**). Interestingly, we observed decreased overall colony radius with increase in the number of doses (**Figure 5A**). While there was no significant improvement from 2 doses to 3 doses, we noticed significant reduction (p<0.003, 2-way ANOVA) in normalized colony radius from one dose to multiple doses. We also noticed increase in the resistant cells’ fractions with the increase in dosing numbers at the final time point (142 hours) (**Figure 5B**), a trend also observed in our mathematical model (**Figure 4D**, top panel). Taken together, our model system can be utilized to identify a lower but effective drug concentration and can be utilized to test which metronomic treatment regimens provide anti-proliferative benefit.

## Discussion

Several clinical trials in the past have shown that the maximum tolerated dose (MTD) approach has not always provided maximum clinical benefits. For example, Cisplatin, a common chemotherapy administered in non-small cell lung cancer patients, failed to show any clinical benefit in terms of overall survival or pathological complete response over relatively moderate doses in a randomized multicenter trial (29). Furthermore, retrospective analyses on low dose metronomic chemotherapy, with frequent schedules proved to be clinically favorable and safer compared to conventional chemotherapy for a large number of drugs in a broad range of tumors (30). In our 2D co-culture system of doxorubicin-sensitive and resistant 4T1 cells, we observed that a cumulative treatment regimen with low dose administered in frequent interval (metronomic chemotherapy or MCT) resulted in the smaller size of colonies as compared with the regimen with a single high dose. This is due to differences in growth rates and drug responses of chemoresistant and chemosensitive cells, which enables optimization with dosing regimens.

In our study, a critical concentration of drug is defined that leads to a colony composition of equal amount of chemoresistant and chemosensitive cells. Here the initial proportion of resistant cells as well as their level of resistance determines this critical concentration. We found the most clinically beneficial timing for therapeutic intervention is when the resistant cell population is less resistant and they are present in lower numbers. Taken together, this 2D co-culture system can be optimized to identify lowest effective doses based on the growth kinetics of chemosensitive and chemoresistant cells in various situations.

The challenges associated with experimentally tracking growth and response of chemosensitive and chemoresistant cells in a mixed culture has inspired mathematical modeling of tumor architectures (31–35). A recent study used an off-lattice agent-based model and flow-cytometry analysis to show the benefits of adaptive therapeutic strategies using dose modulation or treatment vacation in breast cancer patients (19). We took a simplified approach of building our model based on the dose response curves of chemosensitive and chemoresistant cells from the *in vitro* co-culture. Nonetheless, we were able to identify a treatment regimen to attain the smallest sized tumor based on experimental data from an MTT assay that measures ATP produced by live cells after 4 days of incubation with doxorubicin. The specific dose regimens may be different in experiments when normalized colony radius is counted as a measure of viable cells after 7 days, and this can be addressed if dose response curves are created for these assays.

Clinical studies reported that breast cancer relapsed due to drug resistance in 70% of node-positive and 30% of node-negative cases (36–38). However, when head-to-head comparisons are made among the patients, the ratio of sensitive *vs*. resistant cells depend on the time during which diagnostic tests are conducted during the treatment schedule. Thus, in our experimental model we studied 3 main scenarios when the fraction of sensitive cells is higher than that of the resistant cells, vice versa, and when both fractions are equal. As there could possibly be many ratios that are impractical to cover experimentally due to the limit of the throughput, this motivated us to simulate the data for many ratios in our mathematical model.

Heterogeneity, in terms of intratumor cell types and their responses to chemotherapy is a limitation to our study. Using experimental data on dose response curves and cellular growth rates from various cell types in the tumor microenvironment can enable introducing additional parameters to measure the benefit of MCT. In addition, the model also allows us to further optimize it for other cancer types and other chemotherapy and targeted therapy drugs.

As the 2D co-culture system enables tracking of the growth dynamics of chemosensitive and chemoresistant cells under simple epifluorescence microscopy, we can further optimize the system for high throughput screening purposes to identify optimal combinatorial therapies and discovery of new treatment strategies *via* screening of experimental small molecule libraries (39). The 2D co-culture system also provides us the control to build various types of spatial architecture by simply changing the location of initial inoculation. Different architectures quickly created by this model can represent complex patterns of spatial heterogeneity that may vary from one patient to another. Simplified model systems in 2D enabled us to develop initial hypotheses of the clinical situations that would be most beneficial in terms of metronomic therapy dosing regimens. Previous studies showed that response to small molecule inhibitors might differ in 2D *vs*. 3D culture due to difference in model architecture, drug penetration, or cell type (40, 41). In practice, the proposed system 2D system can be used in combination with other assays to account for some of these differences. As a next step, the model could be adapted to account for 3D chemotherapeutic responses with additional parameters, additional cell types, or developed to account for *in vivo* tumor growth rates that correlate with preclinical mouse studies, to eventually develop translatable results to clinical trials.

MCT is currently being studied as a palliative regimen in patients with metastatic breast cancer with the aim to prolong and improve quality of life (42). We envision the use of our model for clinical translation where growth rates and proportions of chemosensitive and chemoresistant cells can be measured to optimize dosing regimens. One possible path is where resistant cells have distinct markers compared to sensitive cells based on the mechanism of resistance, and *in vivo* imaging approaches can quantify their population numbers and growth rates. Another possibility is to perform *ex vivo* expansion of these cell types from patient samples. Then our model could be adapted to account for pharmacokinetics and *in vivo* cell growth rates in tumors, to predict optimal chemotherapeutic regimes. Taken together, with mathematical modeling, the system presented here provides an approach to find optimum therapeutic regimen for heterogenous tumors.

## Data availability statement

The original contributions presented in the study are included in the article/supplementary material. Further inquiries can be directed to the corresponding author.

## Author contributions

DD, SZ, and TD conceptualized the study; SZ, DD, and ML conducted experiments and analyzed the data; TD built the mathematical model and acquired funding; DD, SZ, and TD wrote the manuscript. All authors contributed to the article and approved the submitted version.

## Funding

This study was supported by NIH Pathway to Independence Award (R00CA197649-02).

## Acknowledgments

We thank Oscar Velazquez for transducing 4T1 cells to express Citrine and all the members of the Danino lab for useful discussion. We acknowledge an early preprint of this work on bioRxiv doi: https://doi.org/10.1101/561746 (25).

## Conflict of interest

The authors declare that the research was conducted in the absence of any commercial or financial relationships that could be construed as a potential conflict of interest.

## Publisher’s note

All claims expressed in this article are solely those of the authors and do not necessarily represent those of their affiliated organizations, or those of the publisher, the editors and the reviewers. Any product that may be evaluated in this article, or claim that may be made by its manufacturer, is not guaranteed or endorsed by the publisher.
