# Supplementary material for: Assessing chemotherapy dosing strategies in a spatial cell culture model
Source: Front Oncol. 2022 Nov 24;12:980770. doi: 10.3389/fonc.2022.980770 (PMC9729937; doi:10.3389/fonc.2022.980770)
Supplement: Supplementary file 1 [file DataSheet_1.docx]

Supplementary Material

**Supplementary Figure S1**

**
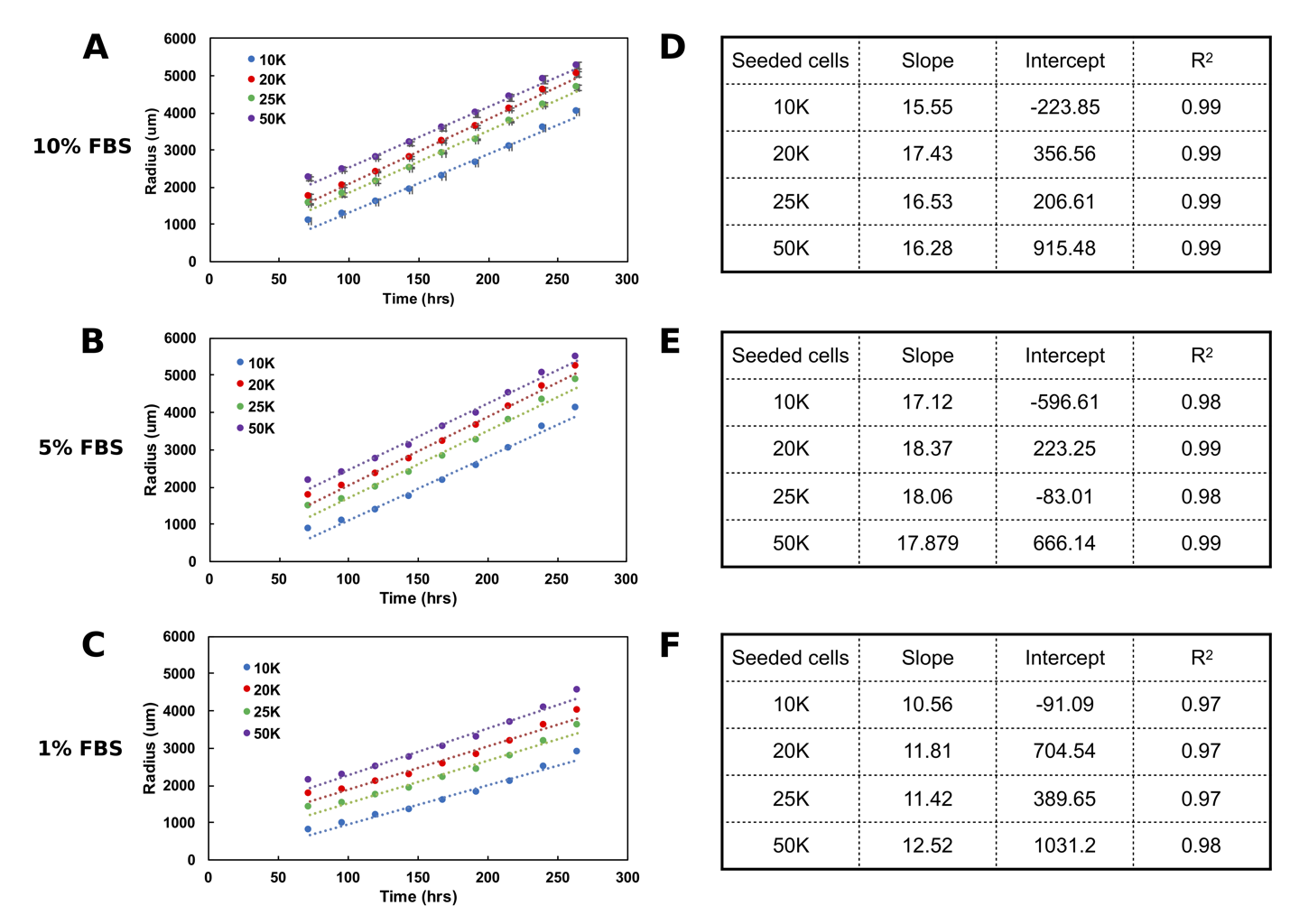
**

**Supplementary Figure S1.** Radial growth dynamics of 4T1-WT monoculture. Colony radius as a function of time for various seeding densities in the presence of (A) 10%, (C) 5%, or (E) 1% FBS. Growth dynamic data were fitted using linear regression, slopes and intercepts of regression lines (dotted) are listed in (B) 10% FBS, (D) 5% FBS, and (F) 1% FBS.

**Supplementary Figure S2**

**
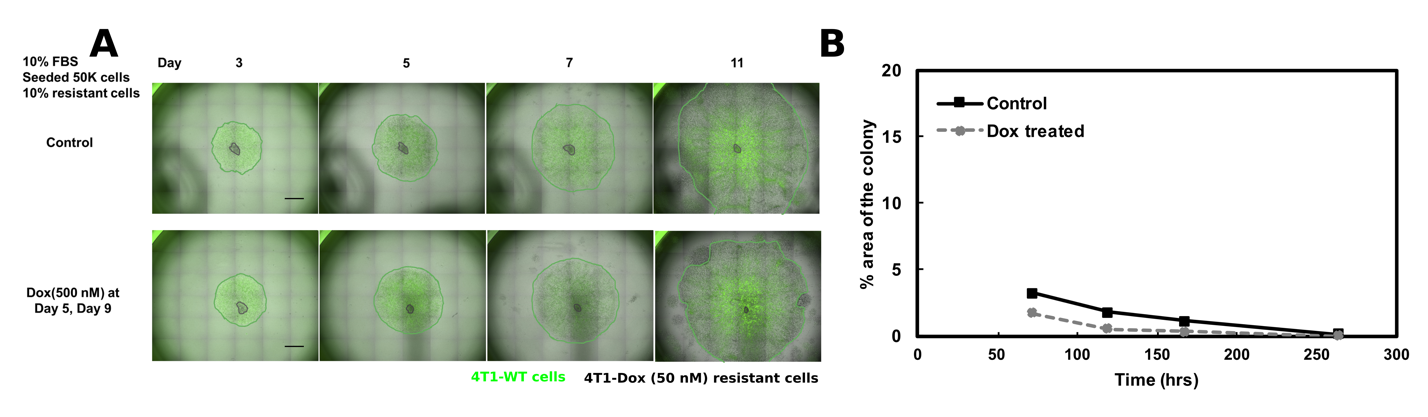
**

**Supplementary Figure S2**. Modulation of spatial competition in colonies where resistant cells were located in the center. (A) Stitched 4X flourescent and bright field composites demonstrating the distribution of chemosensitive cells (4T1-WT, GFP) and 50 nM doxorubicin resistant cells (grey) over time in the presence of 10 % FBS. Colonies were formed by seeding 9:1 of 4T1-WT and chemoresistant cells, chemoresistant cells were seeded in the center of the colony. Colonies were either untreated (top) or treated with two 500 nM doxorubicin doses at day 5 and day 9 (bottom). (B) % area of resistant cells over time in control or doxorubicin treated colonies. Scale bar = 1000 μm.

**Supplementary Figure S3**

**
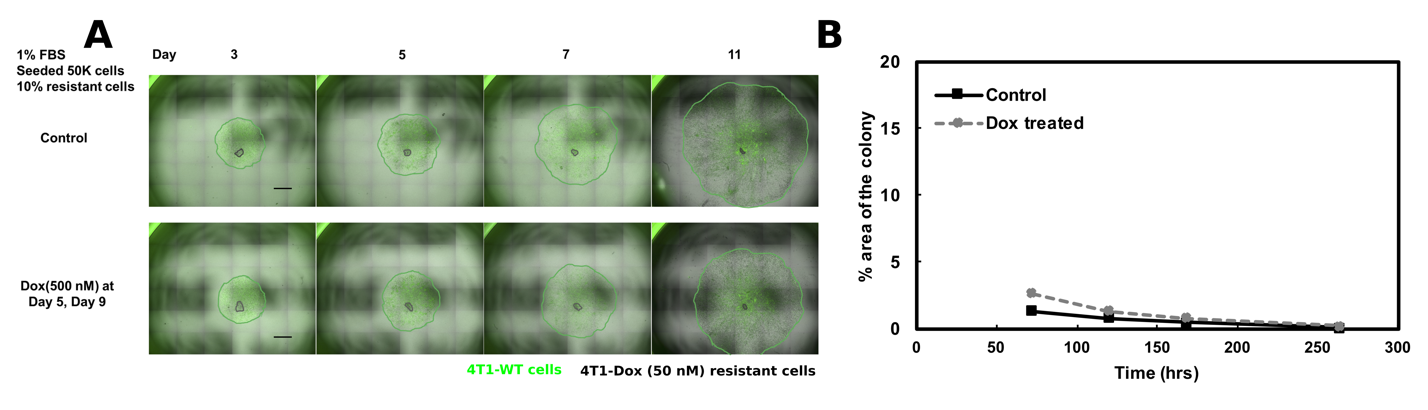
**

**Supplementary Figure S3**. Modulation of spatial competition in colonies where resistant cells were located in the center. (A) Stitched 4X flourescent and bright field composites demonstrating the distribution of chemosenstive cells (4T1-WT, GFP) and 50 nM doxorubicin resistant cells (grey) over time in the presence of 1% FBS. Colonies were formed by seeding 9:1 of 4T1-WT and chemoresistant cells, chemoresistant cell were seeded in the center of the colony. Colonies were either untreated (top) or treated with two 500 nM doxorubicin doses at day 5 and day 9 (bottom). (B) % area of resistant cells over time in control or doxorubicin treated colonies. Scale bar = 1000 μm.

**Supplementary Figure S4**

**
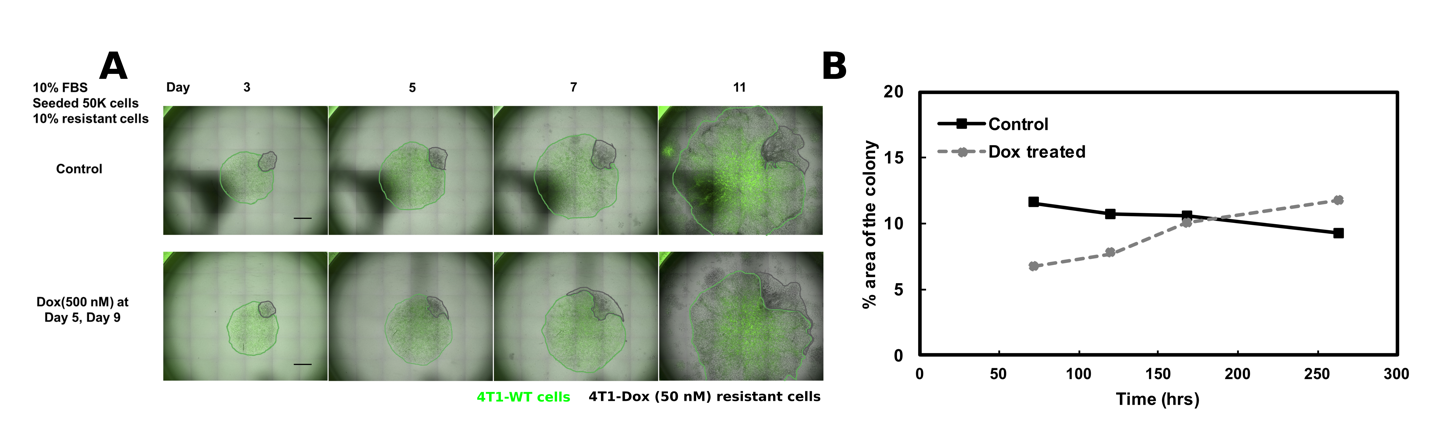
** **Supplementary Figure S4**. Modulation of spatial competition in colonies where resistant cells were located on the edge (A) Stitched 4X flourescent and bright field composites demonstrating the distribution of chemosenstive cells (4T1-WT, GFP) and 50 nM doxorubicin re-sistant cells (grey) over time in the presence of 10% FBS. Colonies were formed by seeding 9:1 of 4T1-WT and chemoresistant cells, chemoresistant cell were seeded on the edge of the colony. Colonies were either untreated (top) or treated with two 500 nM doxorubicin doses at day 5 and day 9 (bottom). (B) % area of resistant cells over time in control or doxorubicin treated colonies. Scale bar = 1000 μm.

**Supplementary Figure S5**

**
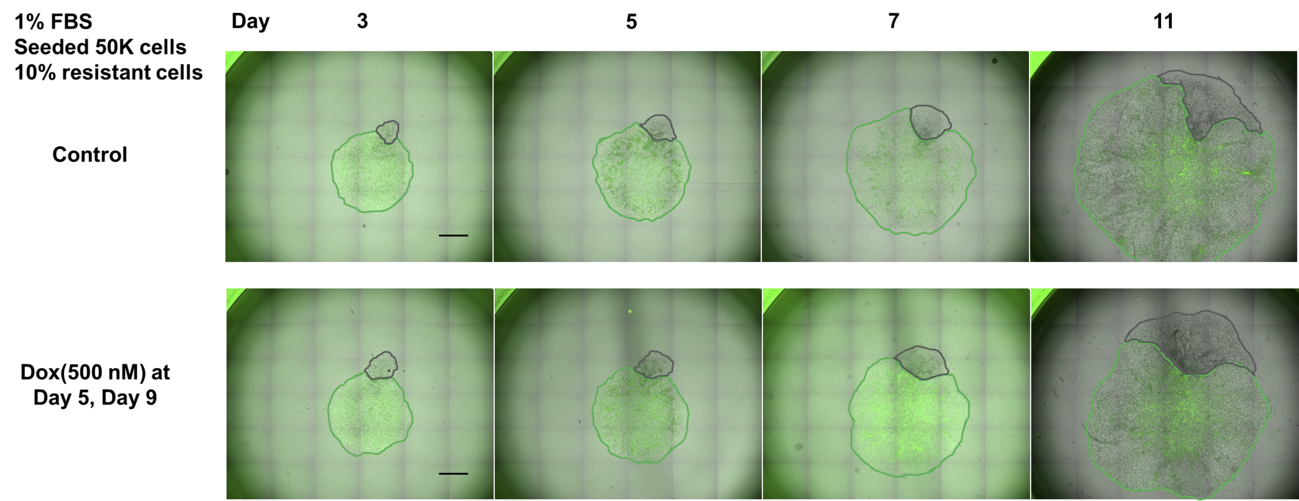
**

**Supplementary Figure S5**. Modulation of spatial competition in colonies where resistant cells were located on the edge (A) Stitched 4X flourescent and bright field composites demonstrating the distribution of chemosenstive cells (4T1-WT, GFP) and 50 nM doxorubicin re-sistant cells (grey) over time in the presence of 1% FBS. Colonies were formed by seeding 9:1 of 4T1-WT and chemoresistant cells, chemoresistant cell were seeded on the edge of the colony. Colonies were either untreated (top) or treated with two 500 nM doxorubicin doses at day 5 and day 9 (bottom). (B) % area of resistant cells over time in control or doxorubicin treated colonies. Scale bar = 1000 μm.

**Supplementary Figure S6**

**Supplementary Figure S6.** Response of 4T1-WildType cells and 4T1-WildType-Citrine cells to doxorubicin treatment as measured by MTT (n=3, error bars represent standard error).

**Supplementary Figure S7**

**
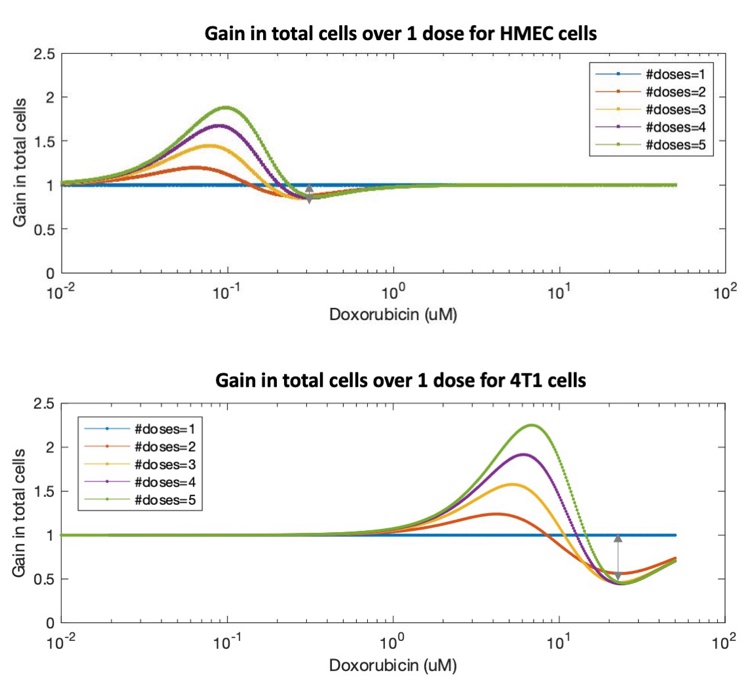
**

**Supplementary Figure S7**. Simulation of collateral effect in human mammary epithelial cells (HMECs). For a 1:1 mixture of Ns (HMEC) to Nr (4T1), number of doses is varied. The ratio of a particular number of doses to 1 dose is termed the “Gain”, for total cell population is plotted. The vertical arrow shows the area of the dose regimen where multiple doses show lower number of total cells over single dose.

**Supplementary Figure S8**

**
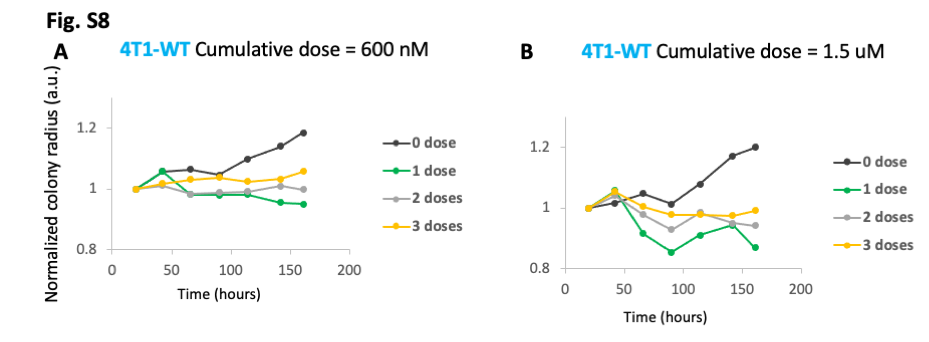
**

**Supplementary Figure S8**. Cumulative treatment regime and long-term overall efficacy. (A) Normalized radius for 3 chemotherapeutic schedules of similar cumulative dose of 600 nM (B) Normalized radius for the same 3 chemotherapeutic schedules of similar cumulative dose of 1.5 μM. Cumulative dosage for each chemotherapeutic schedule is defined as dosage level (nM) multiplied by duration of drug treatment (dosing frequency [1/day] × n days, n = 1, 2 or 3).

**Mathematical model of chemosensitive and chemoresistant cells**

The analytical model for chemosensitive and chemoresistant cell population growth is described in the main text. The model assumes exponential growth of both populations with no competing interactions or limited nutrients. In this simplified model, for each starting dose (x), cells were assumed to change in number given by the fraction of cells viable given in the dose-response curve V(x). For instance, at 2 μM of doxorubicin (Fig 2A), V(2)=0.5, and thus ~50% of WT cells survived for each dose applied. More generally, the number of cells after a single dose of chemotherapy is given by the equation:

N = V(x)*e^a^ (1)

where a is the growth rate and a scaled time interval of t=1 is assumed here for simplicity. In the case of multiple doses, each dose is fractionated across the interval, and the dose is given by X/n, where n is the number of doses. In comparison to a single dose, the total number of cells for multiple doses would be

N = [V(X/n)*e^a/n^]^n^  (2)

This is also equivalent to

N = V(X/n)^n^*e^a^  (3)

Comparing equation 3 with equation 1 shows that the ratio of the number of cells after multiple doses compared to a single dose (termed the gain) is solely dependent on the dose-response curve values and not on growth rate of cells. Here, if V(x) < V (x/n)n, then a single dose will produce less cells (and hence a smaller tumor) than multiple doses. We applied this equation to both chemosensitive and chemoresistant populations by using the experimentally determined Doxorubicin dose response curves in Fig 2A (WT and 4T1 – 800nM).
